# Monomorphic epitheliotropic intestinal T-cell lymphomas: a case report

**DOI:** 10.1186/s13000-021-01143-x

**Published:** 2021-08-30

**Authors:** Haibin Zhong, Yang Zheng, Feiran Zhang

**Affiliations:** Department of General Surgery, The First Affiliated Hospital of Shantou University Medical College, Shantou, 515041 China

**Keywords:** Monomorphic epitheliotropic intestinal T-cell lymphomas, Diagnosis, Prognosis, Case report

## Abstract

**Background:**

Monomorphic epitheliotropic intestinal T-cell lymphomas (MEITL) is a rare and aggressive subtype of lymphoma. The most common site of origin is small intestine. Patients are often presented with diagnosis of intestinal perforation with abdominal pain as the main consulting symptoms. Because of the deficiency of specific diagnostic measures and effective management, diagnosis is often confirmed in advanced stage with poor prognosis.

**Case presentation:**

Here, we introduce a patient who has suffered from abdominal pain and diarrhea, and eventually been diagnosed as Monomorphic epitheliotropic intestinal T-cell lymphomas.

**Conclusion:**

MEITL is rare in clinical practice with deficiency of early diagnostic measures and poor prognosis. Therefore, any patient with ambiguous gastrointestinal symptoms or perforation of the digestive tract where the primary lesion is difficult to identify should be alert to the possibility of this disease.

## Introduction

The gastrointestinal tract is the most common origin of extranodal lymphoma and accounts for 30–40% of all cases. Enteropathy-associated T-cell lymphoma (EATL) is divided into two types: type I EATL and type II EATL. Type I EATL, typically characterized by celiac disease, is common in Western countries. Type II EATL, characterized by abdominal pain and intestinal perforation, is more common in Asia [1]. According to the 2016 WHO classification, type II EATL is formally designated as MEITL which is a primary intestinal T cell lymphoma arising from intestinal intraepithelial lymphocytes [2].

MEITL, a very rare subtype of lymphoma, is involved in less than 5% of lymphoma of the digestive tract. It occurs worldwide with predominance in Asia, and is less common in Western populations. According to the report, the incidence of the elderly is higher with a median age of 58 years in the largest series reported to date [3]. It usually affects the small intestine (jejunum and ileum in particular) and more rarely in duodenum, stomach, and colon [4].

Among all pathological subtypes of lymphoma, B-cell lymphoma is most common which accounts for 90%. In contrast, T-cell lymphoma is rare [5]. Meanwhile, among all the gastrointestinal malignant neoplasms, primary appendicular lymphomas are also rare, with an incidence of only 0.015%.

As for the clinical presentation, the common symptoms of MEITL include abdominal pain, malnutrition, intestinal obstruction, acute abdominal pain, and even intestinal perforation [6]. However, acurate diagnosis is extremely hampered because of the lack of specific clinical symptoms and by the low specificity of the diagnostic approaches. Generally, it needs histopathological analysis of the operative specimen to get the diagnose, especially in the appendiceal neoplasm.

## Case report

A 49-year-old man was admitted to LongHu Hospital, the First Affiliated Hospital of Shantou University Medical College with a history of abdominal pain and diarrhea for 7 days. The pain mainly occurred around the umbilicus, presenting in the manner of colic pain. The patient also complained about generalized fatigue and low-grade fever.

At admission, the body temperature was 38.2 °C, with the pulse 116 beats/min and blood pressure 105/64 mmHg. Physical examination revealed that breath sounds of both lungs were thick and the abdomen was flat with tenderness around the umbilicus, without rebound tenderness or other positive sign.

In history taking, a colonoscopy which revealed ulcer in ascending colon and rectum polyp was performed in the Shantou Central Hospital before admission. Pathological examination revealed chronic inflammation of ascending colonic mucosa accompanied by erosion, and rectal tubular adenoma.

After admission, the patient experienced the pain worsening than before. The pain gradually disseminated to the whole abdomen, presenting persistent colic. Physical examination found tabular abdomen, obvious tenderness and rebound tenderness in the whole abdomen, with reduced bowel sounds. The computed tomography (CT) indicated perforation of digestive tract (most likely in the splenic curvature of the colon) and massive effusion in the abdominal cavity. Therefore, urgent laparoscopy surgery was performed. Intraoperative exploration found no obvious perforation, which could not explain the patient’s peritonitis. After cautious consideration, terminal loop ileostomy was performed in view of potential obscure colonic perforation. With thorough intraoperative exploration, it was found that the serosa of the appendix was crackable and the canal wall was stiff. An appendectomy was performed accordingly and four drainage tubes were placed after thorough abdominal irrigation. The stable postoperative vital signs and gradually reduced drainage fluid indicated smooth recovery.

**Fig. 1:**
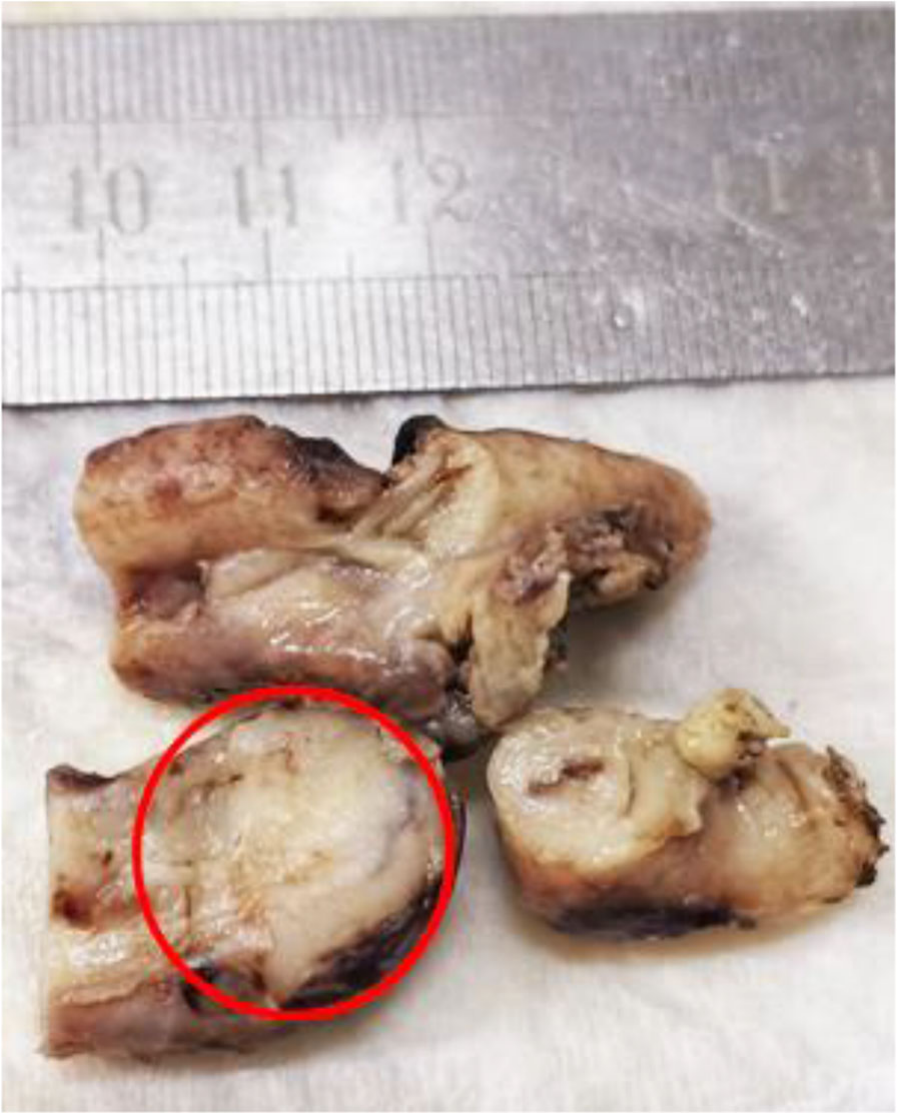
The appendix specimens: stiff texture and luminal occlusion of the appendix blind side

**Fig. 2:**
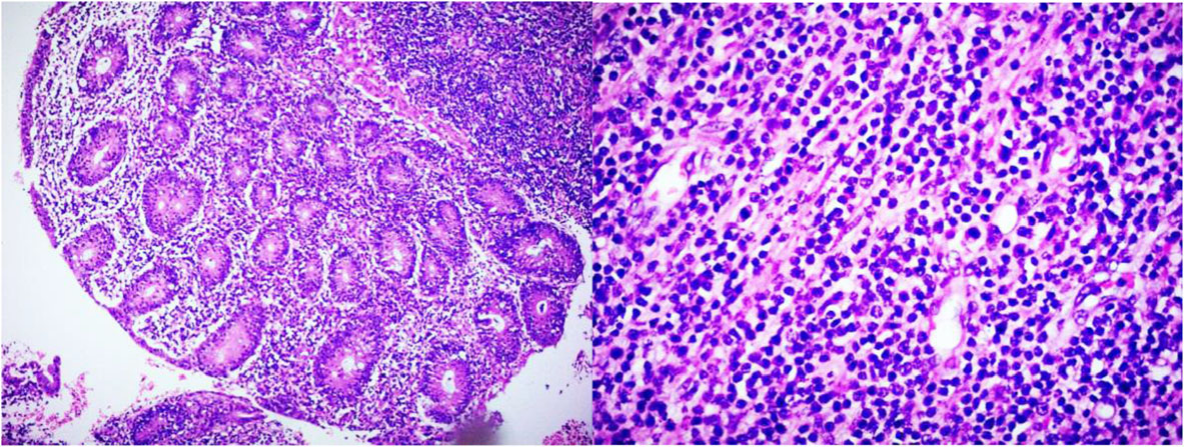
Histological features of the appendix

At histological examination, the diffusely infiltrating lymphocytes at the appendix wall was uniform, with various sizes, coarse-grained chromatin and unclear nucleoli. Epidermophilic phenomena could be seen and lymphoma could not be ruled out (Fig. 1). The microscopic examination found atrophy in some of the glandular duct epithelium with epithelial infiltration of neoplastic lymphocytes in the glandular duct. Tumor cells diffusely distributed throughout the whole wall of the intestine, with single morphology, granular chromatin and obvious nucleoli (Fig. 2).

**Fig. 3:**
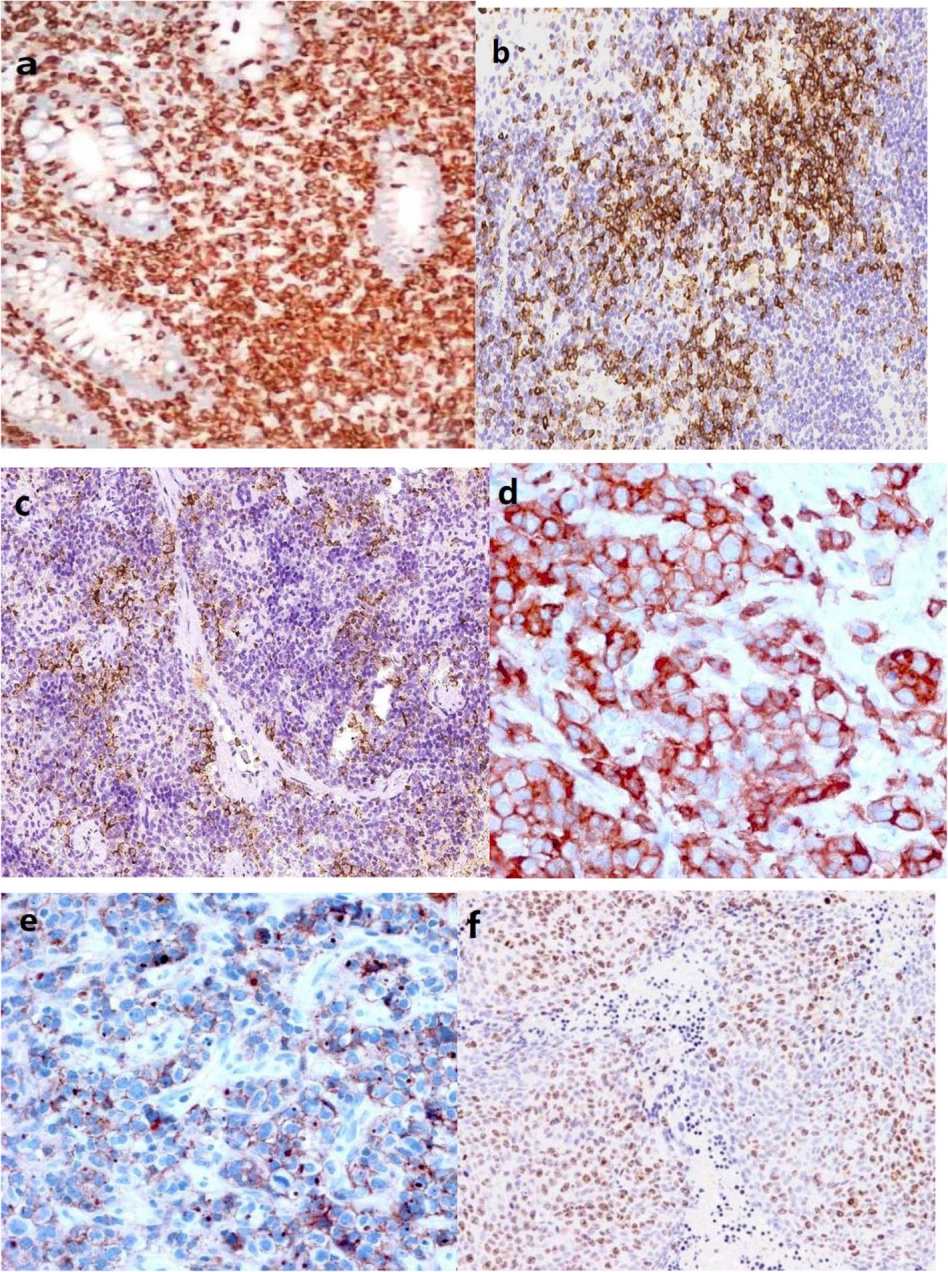
Immunohistochemical positive results, (**a**) for CD3, (**b**) for CD7, (**c**) for CD8, (**d**) for TIA1, (**e**) for CD4, (f) for Ki-67

Immunohistochemistry reveals cell positivity for the following markers: CD3, CD7, CD8, CD56, TIA-1, Ki-67, CD4, while CD5, CD20, CD30, TDT, ALK and EBER are negative (Table 1, Fig. 3).

**Table 1:** Lymphocyte immunophenotype

Lymphocyte immunophenotype	Positive (+) negative (−)
CD3	+
CD7	+
CD8	+
CD56	+
TIA-1	+
CD5	–
CD20	–
CD30	–
TDT	–
ALK	–
EBER	–
CD4	+
Ki-67	+ (80%)

Combined with HE morphological examination and immunohistochemical results, the diagnosis was consistent with MEITL. Moreover, the tumor cells invaded the whole wall of the appendix and the surrounding mesangial adipose tissue.

**Fig. 4:**
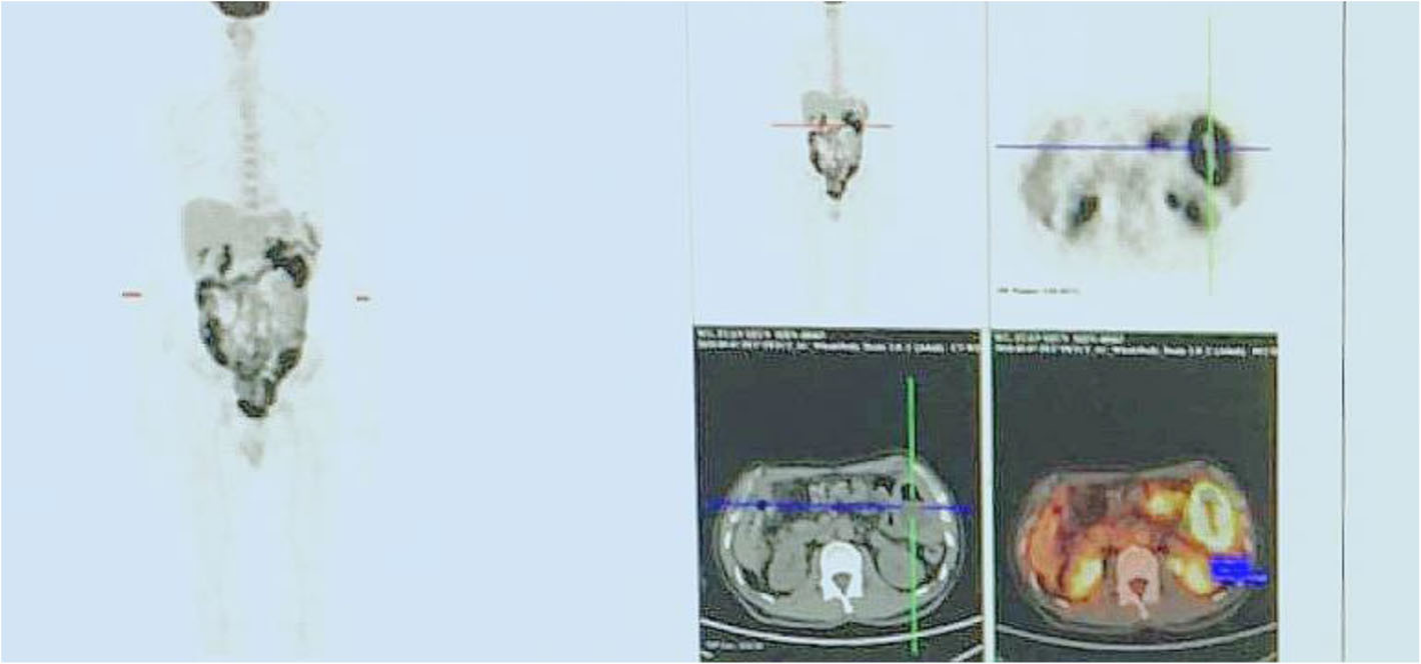
PET-CT scan images

Postoperative reexamination of PET-CT (Fig. 4) indicated thickening in the ileocecal region, colon and stomach, and soft tissue shadow in the lower edge of liver and the outer edge of spleen. In addition, glucose metabolism in the above lesions increased to varying degrees, which was consistent with lymphoma.

Two months after the operation, the patient was admitted to the hospital again due to “abdominal pain and anuria” with primary diagnosis of peritonitis and septic shock. Computed tomography (CT) showed intestinal obstruction with multiple free gases in the abdominal cavity. Abdominal paracentesis and catheterization were performed for drainage and irrigation. The drainage fluid was cloudy dark green with more gas visible in the drainage bags. After treatment with broad-spectrum antibiotics, intravenous fluid resuscitation, analgesics, antiemetics, and antithrombotic drugs, the patient showed no sign of recovery. Subsequently, due to worsening infection, the patient gradually developed thrombocytopenia, coagulation dysfunction, electrolytes imbalance and finally died from multiple organic dysfunction half a month later.

## Discussion

According to published reports, Lymphoma accounts for only a small fraction of all gastrointestinal malignancies with a proportion of about 1–4%. Primary lymphoma of the appendix is rare of all tumors origining from the digestive tract. It presents symptoms of acute appendicitis or acute abdomen.

In this case, the initial symptoms presented were abdominal pain and diarrhea on admission. The patient bore a primary T-cell lymphoma with immunophenotype features consistent with monomorphic epitheliotropic T-cell lymphoma. MEITL, formerly known as type II EATL, is a primary intestinal T-cell lymphoma caused by malignant proliferation of intraepithelial lymphocytes [2]. It is characterized by abdominal pain and intestinal perforation, which is different from type I EATL with classic presentation of celiac disease. Therefore, it is defined as a new subtype of lymphoma in the 2016 WHO classification: MEITL. Even though MEITL is not commonly associated with celiac disease, there are cases of MEITL seen in patients with histologically proven celiac disease [7].

It is reported that small intestine is the most commonly affected part of MEITL, especially jejunum and ileum, and then in order duodenum, stomach, and colon. In our case, the source of the lesion is appendix.

Due to the unspecific clinical manifestations, most cases are not diagnosed until the neoplasm reaches advanced stage. Common laboratory tests and imaging scans were inconclusive. The best approaches to diagnosis are endoscopic biopsy and surgical pathology. Pathological and immunohistochemical results are essential for diagnosis. MEITL is usually positive for CD3, CD8 and CD56, but negative for CD5, CD30 and CD103, with small to medium-sized cells [8]. As in our case, though computed tomography suggested the possibility of gastrointestinal perforation, exploratory laparotomy was fail to find a definite answer to the patient’s peritonitis. We did not get a clear diagnosis until the feedback of the pathological result.

Because of the aggressive progression of the disease, the prognosis of MEITL is generally poor [9]. Therefore, any patient with ambiguous gastrointestinal symptoms or a perforation of the digestive tract where the primary lesion is difficult to identify should be alert to the possibility of this disease, because early diagnosis is essential for timely treatment. We reviewed past literature on MEITL and found that there is still no efficient therapeutic intervention except for stem cell transplant. In general, a variety of combined chemotherapy regimens are used, and autologous stem cell transplantation can also be helpful. However, even with treatment, survival rates remain low. A previous study reported that the median overall survival barely exceeds 1 year (14.8 months) and the median progression free survival is 7 months [10].

## Data Availability

The relevant data and materials pertaining to this study are available upon request.

## Abbreviations

MEITL: Monomorphic epitheliotropic intestinal T-cell lymphomas
EATL: Enteropathy-associated T-cell lymphoma
